# Adult Periodic Alternating Nystagmus Masked by Involuntary Head Movements

**DOI:** 10.3389/fneur.2018.00326

**Published:** 2018-05-14

**Authors:** Diego Kaski, Salman Haider, Amanda Male, Alex Radunovich, Fan Liu, Carla Cordivari, Kailash P. Bhatia, Adolfo M. Bronstein

**Affiliations:** ^1^Department of Neuro-Otology, University College London Hospitals, London, United Kingdom; ^2^Department of Brain Sciences, Imperial College London, London, United Kingdom; ^3^Department of Neurology, Royal London Hospital, London, United Kingdom; ^4^Department of Neurology, National Hospital for Neurology and Neurosurgery, London, United Kingdom

**Keywords:** periodic alternating nystagmus, head tremor, oscillopsia, vestibulo-ocular reflex, rhombencephalitis, psychogenic

## Abstract

Acquired periodic alternating nystagmus (PAN) describes a horizontal jerk nystagmus that reverses its direction with a predictable cycle, and is thought to arise from lesions involving the brainstem and cerebellum. We report a 20-year-old patient with PAN who presented with an acute vertiginous episode and developed an involuntary head movement that initially masked the PAN. The involuntary head movements were abolished with a subtherapeutic dose of botulinum toxin to the neck muscles. We propose that the head movements initially developed as a compensatory movement to the nystagmus, to maintain visual fixation in the presence of the underlying nystagmus, and became an entrained involuntary behavior. This case highlights the importance of disambiguating psychogenic from organic pathology as this may have clinical therapeutic implications, in this case resolution of the most disabling symptom which was her head oscillations, leading to improved day-to-day function despite PAN.

## Introduction

**Figure 1:**
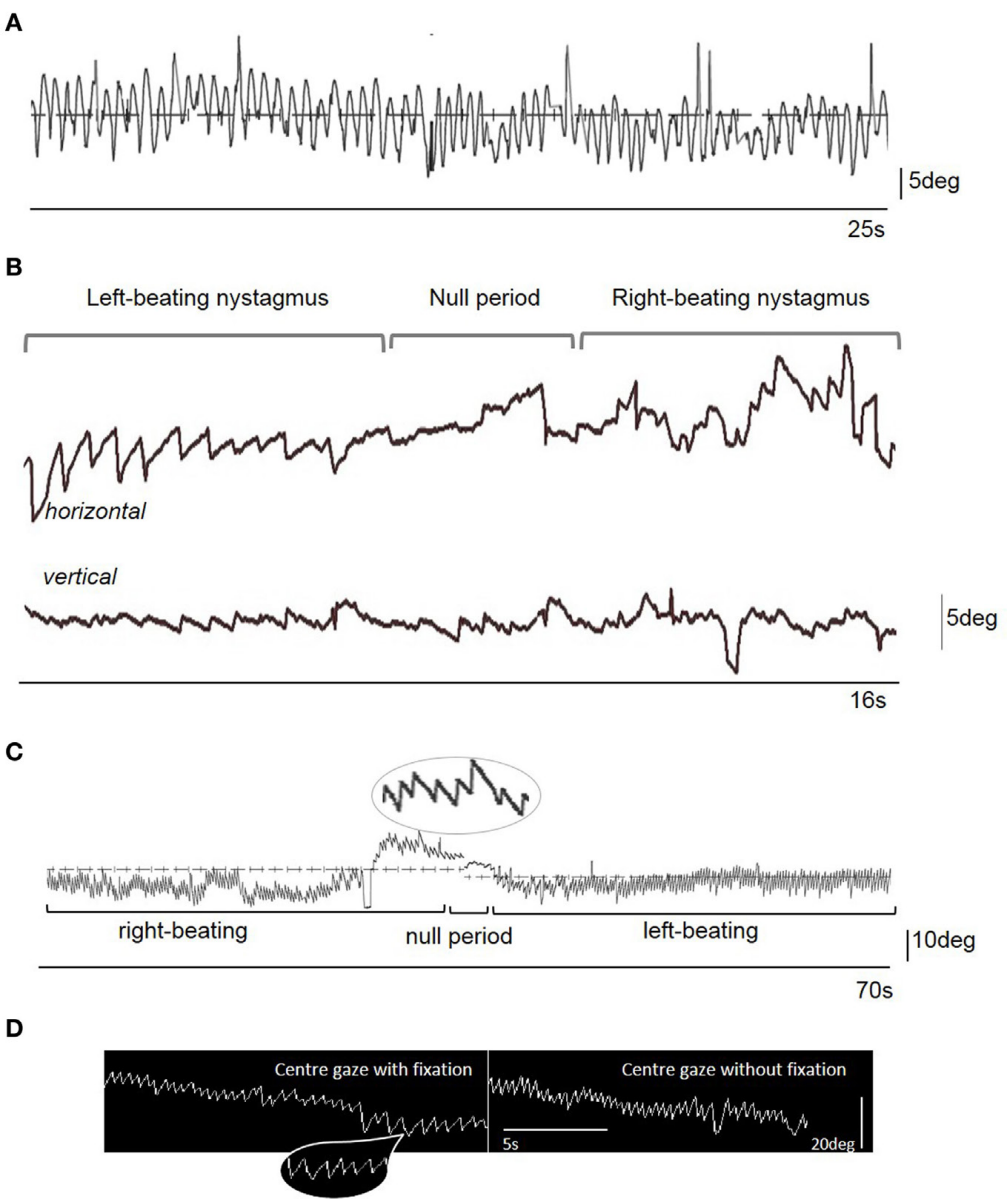
**(A)** Videonystagmographic trace with the patient looking at a fixed central target straight ahead in the light, with the head free taken on Day 13 from symptom onset. Note the sinusoidal eye oscillations representing a compensatory movement to the head oscillations (i.e., a sinusoidal vestibulo-ocular reflex). There is no clear nystagmus evident on the trace (nor on clinical examination). **(B)** Videonystagmographic trace showing periodic alternating nystagmus on day 21 from symptom onset: left-beating predominantly horizontal nystagmus, a null period with no horizontal nystagmus, but prominent vertical (upbeat) nystagmus, followed by a reversal of the horizontal nystagmus into right-beating, with some attenuation of the vertical nystagmus. In central gaze, the left-beating nystagmus peak slow phase eye movement velocity (SPV) was 22°/s, and right-beating SPV was 34°/s. There was upbeat nystagmus starting 5 s prior to the change in nystagmus direction, with a SPV of 10°/s. Upward deflection represents rightward (for horizontal trace), and upward (for vertical trace) eye movements. **(C)** A 70 s videonystagmographic recording of the horizontal eye movements showing a longer period of alternating nystagmus with the eyes in the primary position, without fixation. **(D)** Electronystagmographic recordings in central gaze in the light (with fixation) and the dark (without fixation). Note the mostly linear or decreasing exponential velocity waveform of the nystagmus slow phase [insets in parts **(C,D)**] that is more suggestive of an acquired nystagmus (1). Indeed, no clear congenital-type waveforms were seen in the traces.

A 20-year-old previously well female presented with a history of abrupt onset recurrent episodic (seconds) “dizziness” characterized by a shimmering of the world in front of her. By day 3 she began to experience a constant sensation of vertigo, oscillopsia, mild headache, and prominent vomiting. She was afebrile, had no neck stiffness, but described photophobia. She had gradually developed an involuntary mild side-to-side continuous head movement by day 3. On day 6 there was a constant “no-no” head movement with variable frequency (circa. 2–4 Hz) and amplitude (circa. 15° peak-to-peak) and initially had a fast phase component to the right, with occasional oblique movements (Video S1 in Supplementary Material). The family reported that the movement disappeared during sleep. On admission to hospital, there was right-beating nystagmus (RBN) in the primary position intensifying on right gaze and also apparent on upgaze (Video S1 in Supplementary Material). The intensity of the nystagmus was enhanced during positional maneuvers, without a change in nystagmus direction. Oculographic recordings were not available acutely. The gait was unsteady, with asymmetric step length, inconsistent foot placement, and variable left foot intorsion, suggesting a functional (psychogenic) etiology (Video S2 in Supplementary Material). There was no limb ataxia, and no myoclonus. Ten days after the original assessment the head movements worsened dramatically, rendering interpretation of the eye movements difficult (Figure 1A; Video S3 in Supplementary Material), but the patient continued to complain of oscillopsia, even when the examiner attempted to restrain the head manually (the patient did not consent to the use of a bite bar). The head tremor resolved immediately on day 21 after symptom onset with subtherapeutic 50 mouse units of abobotulinumtoxin A applied to the levator scapulae muscles bilaterally. With the head now still it became possible to visualize the eye movements revealing periodic alternating nystagmus (PAN) with a cycle of approximately 90 s, and a null phase of 2 s (Figures 1B,C; Video S4 in Supplementary Material).

**Figure 2:**
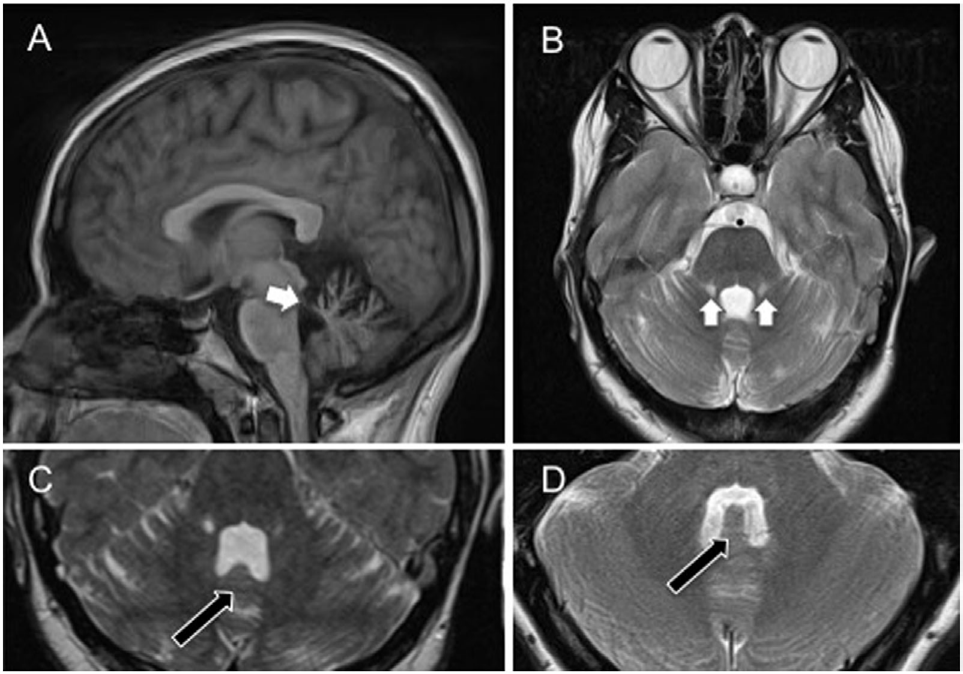
**(A)** Sagittal cross-sectional T1-weighted magnetic resonance image showing atrophy of the superior cerebellar peduncles, and anterior aspects of the cerebellum (white arrow) involving the uvula and nodulus. **(B)** Coronal T2-weighted magnetic resonance image showing bilateral atrophy of the anterior cerebellum, including the nodulus and uvula, and superior cerebellar peduncles with associated high signal (gliotic) change. **(C)** Axial T2-weighted magnetic resonance image showing atrophy of the nodulus (black arrow) within the enlarged fourth ventricle. **(D)** Axial T2-weighted magnetic resonance image showing nodulus (black arrow) and fourth ventricle in a healthy age-matched control. Note the flattening of the nodulus and expansion of the fourth ventricle in **(C)**.

An MRI brain scan on day 6 showed bilateral symmetrical superior cerebellar peduncle atrophy, thinning of the anterior cerebellar structures, and enlargement of the fourth ventricle (Figure 2), unchanged at 6-month follow-up. Initial cerebrospinal fluid (CSF) analysis revealed normal protein and no cells. Oligoclonal bands were absent in CSF and serum, and full viral PCR negative in the CSF. A vasculitic and paraneoplastic screen, whole body PET-CT, spinocerebellar ataxia 1, 2, 3, 6, 7, 8, and 12 gene testing, and autoimmune screen including anti-GAD and anti-Gq1b antibodies were normal. Pure tone audiometry on day 13 was normal, and vestibular responses to impulse chair rotation, taking into consideration the involuntary head oscillations, were normal and symmetrical (also day 13).

She was treated with baclofen titrated up to 90 mg three times daily. There was no objective reduction in the slow phase velocity (SPV) of the eye movements (Table 1), despite symptomatic improvement in vision and balance. Given ongoing oscillopsia, gabapentin was added to the baclofen with no improvement. A trial of clonazepam caused drowsiness and was discontinued. The patient improved following a 10-month course of vestibular rehabilitation focusing on sensory integration, functional gait, postural muscle strengthening, increasing physical activities, and maximizing her independence. She was referred for cognitive behavioral therapy and underwent psychological counseling. The patient’s condition plateaued, she commenced a university course, and was able to navigate familiar environments.

**Table 1:** Peak slow phase velocity (SPV) of the right-beating nystagmus (RBN) and left-beating nystagmus (LBN) component of the periodic alternating nystagmus before and after treatment with baclofen 90 mg three times daily.

Date of recording from symptom onset	RBN SPV (°/s)	LBN SPV (°/s)
Day 21	34	22
Day 94	33	23

*SPV was measured using videooculography, and with the patient looking in the primary position, in the dark*.

## Background

Acquired PAN refers to a horizontal jerk nystagmus that reverses its direction every 90–120 s with a brief transition (null) period (2). PAN is thought to arise from an unstable “velocity-storage mechanism”—a neural brainstem network that prolongs the duration of peripheral vestibular afferent signals from the semicircular canals (2). PAN is most often described in its infantile form (3–6), although acquired PAN has been associated with demyelinating disease (7, 8), posterior fossa malformation (3), spinocerebellar degeneration (9–11), and anticonvulsant medications (12, 13); in many cases the cause is never identified. There have been reported cases of PAN as a direct consequence of visual (14) or vestibular (15) loss, as a transient consequence of sensory deprivation (6). This is to our knowledge the first description of PAN emerging in adulthood following an acute vertiginous episode that was masked by an associated head tremor.

## Discussion

We report a case of PAN following an acute episode of vertigo and oscillopsia masked by the presence of a vigorous head oscillation (Figure 1C). Oculographic recordings were not available on presentation and we cannot, therefore, conclusively rule out the presence of PAN acutely. Nevertheless, the clinical oculomotor examination revealed RBN in the primary position intensifying on right gaze and also apparent on upgaze suggesting either an acute vestibular insult (not apparent, however, on objective vestibular testing), or the early presence of PAN. The intensity of the head oscillations masked the PAN at this stage, which later became apparent as the head tremor abated. Given the negative viral CSF PCR, negative paraneoplastic screen, and serial imaging performed showing stable appearances of the symmetrical superior cerebellar peduncle atrophy, the cause of the acute vertiginous episode remains unclear. Chiu and Hain (15) described unmasking of PAN in a patient with underlying cerebellar atrophy due to an acute attack of Meniere’s. Given superior cerebellar peduncle atrophy apparent within the first few days of symptom onset, and thus unlikely to have developed abruptly, a similar explanation for the PAN being unmasked due to an acute illness is possible. Our patient, however, had only a single vertiginous episode and did not manifest any hearing loss on audiograms or vestibular loss on impulse chair rotation testing to suggest a peripheral vestibulopathy. The cerebellar atrophy may have thus reflected more widespread (possibly congenital) cerebellar development that predisposed the patient to PAN following an acute illness. Congenital-type nystagmus emerging in adulthood, without a clear precipitant, has been previously described (16). The absence of other congenital nystagmus features (Figures 1C,D), that she had had several optometrist appointments throughout her life without anyone commenting on nystagmus, and that her family members had never observed any nystagmus prior to this episode, argue against the PAN having been manifest prior to this illness. Although baclofen can abolish nystagmus and improve oscillopsia in acquired PAN (17), this is not a universal finding and tends to be less effective in congenital forms (18). The lack of efficacy of baclofen may lend support to the proposed long-standing cerebellar atrophy underpinning the predisposition to PAN in this patient.

This is to our knowledge the first report of PAN masked by an involuntary head movement. The head movement was noted to be gradual in onset and progressive, initially appearing to “beat” to the left, and later became multidirectional (Video S3 in Supplementary Material). One possibility is that the head movement developed as a compensatory movement to the nystagmus, to maintain visual fixation in light of the underlying nystagmus that became an entrained involuntary behavior (i.e., functional/psychogenic). Head movements that are compensatory to nystagmus are a characteristic feature of spasmus nutans—a rare condition of childhood characterized by asymmetric pendular nystagmus, head nodding, and torticollis (19). In our patient, however, the head tremor was abolished immediately following subtherapeutic doses of botulinum toxin A, suggesting a functional etiology (20). Indeed, the lack of “periodicity” of the head movement, and the jerky nature, argue in favor of a functional head movement rather than a compensatory behavior.

## Concluding Remarks

In summary (i) we describe the co-existence of a clinically visible head oscillation with adult-onset PAN; (ii) the possible clinical masking of the PAN as a consequence of the head oscillations; and (iii) the emergence of PAN in adult life consequent upon an acute vertiginous illness of unknown origin. From a practical perspective, this case highlights the importance of disambiguating psychogenic from organic pathology as this may have clinical therapeutic implications, in this case resolution of the most disabling symptom which was her head tremor, leading to improved day-to-day function despite PAN. Distractability, disappearance of the tremor or entrainability during a concurrent motor task or during the *casual* examination (21) are all features of a functional (psychogenic) tremor but were absent in this patient. The multi-planar and large amplitude oscillations of the head, together with the absence of a recognized association between PAN and head tremor led us to suspect a psychogenic origin, and treatment with subtherapeutic botulinum toxin.

## Ethics Statement

Written informed consent was obtained from the patient for publication of the case history and videos. Ethics approval was not required.

## Author Contributions

DK performed the clinical assessments, compiled the manuscript and figures, and approved the final version. SH, AM, AR, FL, CC, and KB reviewed the patient and performed the clinical assessments. AB reviewed the patient and approved the final version of the manuscript.

## Conflict of Interest Statement

The authors declare that the research was conducted in the absence of any commercial or financial relationships that could be construed as a potential conflict of interest.

## Supplementary Material

Video S1 Video recording 6 days after symptom onset showing right-beating nystagmus in the primary position intensifying on right gaze and also apparent on upgaze. There were occasional erratic saccadic eye movements that the patient related to her visual disturbance, seen mostly in the video when the patient is looking to the left. Note the side-to-side head tremor, best seen toward the end of the video. The examiner is attempting to restrain the head manually to facilitate eye movement assessment. NB the patient was myopic (−3.5 diopters right, −5.0 diopters left), and yearly optometric assessments had never revealed nystagmus nor latent nystagmus. Visual acuity prior to the detection of periodic alternating nystagmus but in the context of involuntary head movements was 6/12 [right eye (RE)] and 6/12-2 [left eye (LE)]. Once the head movements had abated, visual acuities were 6/36 (RE) and 6/36 (LE).

Video S2 Gait assessment 13 days after symptom onset showing unsteadiness, a variable stance width, asymmetric stride length, and lurching movements. The patient reported marked unsteadiness on turning and was unable to perform tandem gait.

Video S3 Head tremor 13 days after symptom onset. The examiner standing behind the patient is attempting to restrain the head manually to facilitate eye movement assessment. Note the marked mostly horizontal (with some oblique movements) tremor of the head. This rendered the interpretation of the eye movements extremely difficult. The patient was unable to abate the head tremor or alter the amplitude of these movements to command, but instead the tremor became more marked when trying to restrain the head manually.

Video S4 Eye and head movements on day 21, 30 min after subtherapeutic botulinum toxin application to the neck muscles.
